# Oxidative Stress and Arterial Dysfunction in Peripheral Artery Disease

**DOI:** 10.3390/antiox7100145

**Published:** 2018-10-19

**Authors:** Ahmed Ismaeel, Robert S. Brumberg, Jeffrey S. Kirk, Evlampia Papoutsi, Patrick J. Farmer, William T. Bohannon, Robert S. Smith, Jack L. Eidson, Ian Sawicki, Panagiotis Koutakis

**Affiliations:** 1 Department of Nutrition, Food and Exercise Sciences, Florida State University, Tallahassee, FL 32304, USA; ai18@my.fsu.edu (A.I.); epapoutsi@fsu.edu (E.P.); 2 Department of Surgery, Vascular Surgery Associates, Florida State University School of Medicine, Tallahassee Memorial Hospital, Tallahassee, FL 32308, USA; rbrumberg@vsafl.com; 3 Department of Surgery, Capital Regional Medical Center, Tallahassee, FL 32308, USA; jeffrey.kirk@hcahealthcare.com; 4 Department of Chemistry and Biochemistry, Baylor University, Waco, TX 76798, USA; patrick_farmer@baylor.edu; 5 Department of Surgery, Baylor Scott and White Medical Center, Temple, TX 76508, USA; william.bohannon@bswhealth.org (W.T.B.); robert.smith@bswhealth.org (R.S.S.); jack.eidson@bswhealth.org (J.L.E.); ian.sawicki@bswhealth.org (I.S.)

**Keywords:** endothelial dysfunction, arterial stiffness, inflammation, NOX2, NO

## Abstract

Peripheral artery disease (PAD) is an atherosclerotic disease characterized by a narrowing of the arteries in the lower extremities. Disease manifestations are the result of more than just reduced blood flow, and include endothelial dysfunction, arterial stiffness, and inflammation. Growing evidence suggests that these factors lead to functional impairment and decline in PAD patients. Oxidative stress also plays an important role in the disease, and a growing amount of data suggest a link between arterial dysfunction and oxidative stress. In this review, we present the current evidence for the involvement of endothelial dysfunction, arterial stiffness, and inflammation in the pathophysiology of PAD. We also discuss the links between these factors and oxidative stress, with a focus on nicotinamide adenine dinucleotide phosphate (NADPH) oxidase 2 (NOX2)-derived reactive oxygen species (ROS) and decreased nitric oxide (NO) bioavailability. Finally, the potential therapeutic role of NOX2 antioxidants for improving arterial function and functional status in PAD patients is explored.

## 1. Introduction

Peripheral artery disease (PAD) is an atherosclerotic disease characterized by a narrowing of the arteries in the lower extremities [1]. PAD may be asymptomatic or symptomatic, and the spectrum of symptoms is classified according to the Rutherford classification [2]. Stage 0 is considered asymptomatic, and patients presenting with mild or moderate claudication, or walking-induced leg muscle pain relieved by rest, are classified in Stages 1 and 2, respectively. In the later stages of PAD, patients exhibit foot pain at rest (Stage 4), and/or ulcers and gangrene (Stages 5 and 6) [2].

While ischemia is a major operating mechanism in PAD, the disease manifestations are due to more than just reduced blood flow. For example, there is a clear myopathy present in skeletal muscle of PAD patients, which is characterized by abnormal skeletal muscle function and morphology, as well as metabolic defects [3]. The walking impairment in PAD is believed to involve these abnormal muscle characteristics, in addition to impaired hemodynamics, arterial stiffness, endothelial dysfunction, and inflammation [4]. 

An important tool for screening for PAD is the ankle-brachial pressure index (ABI), which is the ratio of the blood pressure at the ankle to the blood pressure in the upper arm [5]. Normal ABI ranges from 1.0 to 1.4, and values below 0.9 are considered diagnostic of PAD [5]. Traditionally, the ABI has been considered the best prognostic indicator in PAD [5]. However, the available evidence does not seem to support a major role for hemodynamics in determining functional capacity. For example, a study found no relationship between ABI and walking capacity in a group of PAD patients [6]. Likewise, another study found no relationship between ABI and either maximum walking distance (MWD) or pain-free walking distance (PFWD) [7]. In contrast, measures of endothelial function and arterial stiffness are associated with exercise performance in PAD [8]. Therefore, it has been suggested that decreased functional status in PAD patients may be the result of other mechanisms, including arterial dysfunction and inflammation, and that further investigation is required to identify the mechanisms underlying these associations [8]. 

Oxidative stress plays an important role in PAD and is believed to contribute to complications and disease progression [9]. Enhanced production of reactive oxygen species (ROS) may be involved in the pathophysiological disabilities associated with PAD, including decreased walking distance and quality of life [10]. Furthermore, oxidative stress is recognized as a major determinant of endothelial dysfunction and vascular inflammation [11]. The main purpose of this review is to present the current evidence for the involvement of endothelial dysfunction, arterial stiffness, and inflammation in the pathophysiology of PAD. We also discuss the links between these processes and oxidative stress, with a focus on the mechanistic role of NADPH oxidase 2 (NOX2). Finally, we present the possible therapeutic role of antioxidants for improving the arterial function of PAD patients. 

## 2. Endothelial Dysfunction

Endothelial dysfunction broadly refers to a diminished production or bioavailability of nitric oxide (NO) as well as imbalanced vasorelaxant and vasoconstrictor substances. It is known to precede atherosclerotic development and is involved in lesion formation by up-regulating adhesion molecules, increasing inflammation and cell permeability, increasing low-density lipoprotein (LDL) oxidation, and increasing smooth muscle cell proliferation and migration [12]. Since endothelial dysfunction is a major player in atherogenesis, measurements of endothelial function have been implicated as markers of cardiovascular risk in PAD. One common technique used to measure endothelial function is flow-mediated dilation (FMD), which assesses the change in brachial artery diameter due to reactive hyperemia following a brief period of ischemia using ultrasound [13]. FMD is expressed as the percent change in diameter from baseline, and the measure is correlated with endothelial function [13].

A low FMD has been shown to be an independent predictor of cardiovascular risk in patients with PAD. In fact, a low FMD is associated with a greater risk of leg amputation as well as coronary heart disease [14,15]. Interestingly, however, there do not seem to be significant differences in FMD according to the severity of PAD [16]. This suggests that endothelial dysfunction may be a process that begins in the early stages of the disease. More recently, endothelial dysfunction assessed by another non-invasive test for evaluating endothelial function, peripheral artery tonometry (PAT), has also been shown to correlate with a history of cerebrovascular disease [17]. PAT is assessed at the fingertip using probes with inflatable cuffs, and the ratio of reactive hyperemia response to basal flow is calculated [18]. 

FMD is believed to be mediated by NO [13]. We have recently reviewed the evidence for elevated oxidative stress in PAD, as well as the interplay between NO and oxidative stress [19]. High levels of ROS can lead to the rapid inactivation of NO to form peroxynitrite, thus reducing the amount of bioactive NO [20]. Notably, peroxynitrite is a strong oxidant molecule that contributes to atherogenesis. Furthermore, peroxynitrite can uncouple endothelial nitric oxide synthase (eNOS), thus resulting in increased superoxide and decreased NO production [20]. Due to this relationship, there is a strong rationale for oxidative stress inducing the arterial dysfunction observed in PAD via reduced NO bioactivity, and studies have shown that oxidative stress is implicated in reduced FMD in PAD patients. For example, PAD patients were shown to have elevated serum levels of the oxidative stress marker 8-Hydroxy-2-deoxy-2-deoxyguanosine (8-OHdG), a compound produced by oxidative damage of DNA by ROS, as well as reduced serum levels of nitrite and nitrate (NOx) and lowered FMD, compared with controls [21]. The sum of nitrite and nitrate is commonly used to quantify NO bioavailability in vivo [22]. NOx was correlated with both FMD and 8-OHdG, suggesting that oxidative stress may be involved in reducing NO bioavailability, and therefore arterial dysfunction [21]. 

## 3. NADPH Oxidase 2 (NOX2)

NADPH oxidase (NOX) enzymes are enzymes with the main purpose of producing the ROS superoxide and H_2_O_2_ by catalyzing the transfer of electrons from NADPH in the cytosol to molecular O_2_ [23]. NOX enzymes are present in the vascular wall and are the most important source of superoxide in vascular cells. However, these ROS are normally produced at low, managed levels that are important for normal physiological function, playing a role in innate immunity, redox signaling during metabolism, and as cofactors in hormone production [24]. The catalytic core of NOX, gp91 (phox), is a membrane-bound subunit that is inactive until it binds to membrane-anchored p22phox, which stabilizes the catalytic subunit in the plasma or intracellular membrane. Upon activation, the regulatory subunits p67phox, which induces conformational changes that facilitate electron flow, p47phox, which acts as an organizer protein and chaperones p67phox to the membrane, p40phox, important for ROS production, and Rac guanosine triphosphatase (GTPase), which assists in membrane tethering, are recruited to form the active enzyme complex on the membrane [23]. Historically, only underactive NOX systems were of interest, as this leads to the rare condition known as chronic granulomatous disease, in which the ability of leukocytes to produce ROS is impaired, which causes a greater susceptibility to microbial infections [24]. More recently, however, evidence has emerged implicating overactive NOX systems in the initiation and progression of vascular disease via excessive ROS production by cells of the artery wall in levels that are cytotoxic [24]. These ROS may lead to activation of pro-inflammatory pathways, depletion of antioxidants, and oxidative damage to proteins, lipids, and DNA [23]. Furthermore, NOX-derived ROS can promote the oxidation of cysteine residues within xanthine dehydrogenase enzymes, forming the enzyme xanthine oxidase, which uses O_2_ as the terminal electron acceptor, producing more ROS [23]. 

Of the NOX isoforms, NOX2 is believed to have the greatest implication in vascular disease. Culturing of endothelial cells with oxidized LDL induces increased NOX2 expression and ROS formation, but NOX2 inhibition prevents the release of ROS [25]. Likewise, overexpression of NOX2 in mice results in significantly increased superoxide production [26,27], and *Nox2* knockout mice show significantly reduced ROS levels [28,29]. In humans, a hereditary deficiency of the gp91 (phox), is also associated with decreased markers of oxidative stress [30]. Therefore, increased activation of NOX2 is believed to contribute to diminished bioavailability of NO, and thus, endothelial dysfunction and vascular cell hypertrophy [31]. NOX2 up-regulation could also explain the oxidative stress observed in PAD patients and account for endothelial dysfunction. To test this hypothesis, the interplay among FMD, NOX2, and oxidative stress was analyzed in PAD patients and matched controls [32]. Patients with PAD had greater NOX2 activation and increased levels of serum isoprostanes, which are produced by free-radical catalyzed peroxidation of arachidonic acid, and often considered the “gold standard” for lipid peroxidation and oxidative stress quantification in vivo. Furthermore, NOx and FMD were reduced in PAD patients compared to controls, and FMD was independently associated with NOX2 activation [32]. 

## 4. Arterial Stiffness

Another measure of arterial function is arterial stiffness, which is largely determined by the ratio of elastin to collagen in arterial walls. Aging and atherosclerosis can lead to arterial stiffness that is due to elastic fiber degeneration and greater collagen fiber recruitment [33]. The pulse wave across an artery has a velocity that depends on the stiffness of the arterial segment, and because of the high resistance of arterioles of the periphery, there is an impedance mismatch which leads to the generation of a retrograde wave. In the case of increased arterial stiffness, the velocity of the pulse wave propagation increases, and the pulse wave travels quicker and gets reflected sooner, returning to the ascending aorta during systole, which leads to augmented systolic blood pressure [34]. Therefore, aortic pulse wave velocity (aPWV) can be used to determine aortic stiffness, and in PAD patients, aPWV has been shown to be higher compared with controls [35]. Additionally, the aortic augmentation index (AIX), the difference between the second and first systolic peaks in the aortic pressure waveform, is a measure of pulse wave reflection, or more specifically, the contribution of the reflected pulse wave to central pulse pressure [34]. While AIX is dependent on arterial stiffness and the reflective properties of arteries and is correlated with aPWV, the two measurements are not interchangeable, as AIX is not a true indicator of arterial stiffness, but rather an index of wave reflection, including aPWV, and is also affected by heart rate and height [36,37,38]. Notably, AIX is associated with known risk factors of cardiovascular disease as well as death from cardiovascular disease and is significantly correlated with degree of coronary artery disease [36]. PAD patients have also been shown to have higher central and peripheral AIX compared with controls, and in fact, each 10-unit increase in central AIX and peripheral AIX was associated with significantly increased odds of PAD [39]. 

An increase in aPWV by just 1 m/s has been estimated to increase risk of cardiovascular events by 14% [40]. Several mechanisms have been proposed to explain this phenomenon. For example, arterial stiffness is related to lower diastolic blood pressure in the ascending aorta, which can lead to low perfusion pressure and reduced coronary perfusion. However, arterial stiffness is also related to higher systolic blood pressure as well; together, these factors can lead to increased oxygen demand of the myocardium that is not met, which can result in ischemia [41]. Likewise, increased aPWV and central arterial pressure due to arterial stiffness can lead to higher arterial pulsatility, which can damage the microcirculation, especially in highly perfused organs such as the heart [41]. Finally, as the stiffness of an artery increases, the hemodynamic load to which the artery’s endothelium is exposed becomes greater, which can result in greater damage of the endothelium [41]. 

Arterial stiffness is believed to be linked with oxidative stress in several disorders by uncoupling of NOS, and by oxidative damage to the proteins, lipids, and DNA of vascular endothelial cells [42]. Inflammation may also lead to arterial stiffness, as inflammatory cytokines can play a role in decreased smooth muscle cell relaxation by reducing NO bioavailability and increasing levels of the vasoconstrictor endothelin-1 [43]. On the other hand, oxidative stress and inflammation may also lead to structural arterial stiffening by stimulating hyperplasia of vascular smooth muscle cells and increased collagen synthesis [43]. Specifically, inflammatory cytokines can lead to leukocyte infiltration in blood vessels, which may cause the release of matrix metalloproteinases, enhancing elastin degradation and increasing the generation of uncoiled, stiff collagen [43]. Finally, increased expression of osteoblast markers by smooth muscle cells in chronic inflammatory conditions can lead to stiffening of the extracellular matrix, calcification, and reduced elasticity of vessels [43]. 

A higher AIX and aPWV has been shown to be correlated with 8-iso-prostaglandin F2a (F2-IsoPs), an indicator of oxidative stress, only in PAD patients and not in controls [44]. Likewise, in a metabolic profiling of PAD patients, oxidized LDL was independently associated with the aPWV, suggesting that arterial stiffening may be determined by the level of oxidative modifications [45]. Furthermore, arterial elasticity is a measure of vascular compliance and distensibility in addition to arterial stiffness; it can be quantified based on a modified Windkessel model, with C1 being a marker for large artery elasticity and C2 a marker for small artery elasticity [46]. Decreased artery elasticity has been identified as an independent predictor of all-cause and cardiovascular mortality in patients with PAD [47]. Additionally, an inverse association has been reported between C1 and F2-IsoPs as well as C2 and F2-IsoPs in PAD patients but not in controls, providing further evidence that oxidative modifications may be involved in altered arterial elasticity in PAD [48]. 

## 5. Inflammation

Atherosclerosis was first referred to as an inflammatory disease as early as 1999 [49]. It is now understood that atherosclerosis is more than just a disease of plaque deposition, but rather an interaction of endothelial dysfunction, inflammation, and classical risk factors which lead to progressive arterial damage and disease [50]. Large prospective studies including the Physician’s Health Study and the Edinburgh Artery Study have found that the relative risk of developing PAD is independently associated with different inflammatory markers including C-reactive protein (CRP) and intercellular adhesion molecule-1 (ICAM-1) [51,52]. Notably, CRP has been extensively studied as a marker of PAD, and shown to be inversely related to ABI values and to the success of angioplasty procedures [53,54]. However, in addition to its role as a potential biomarker of inflammation and disease, CRP is also implicated in disease progression as well. For example, CRP leads to the release of several pro-inflammatory cytokines and induces release of endothelial monocyte chemoattractor protein-1 (MCP-1), which is a chemokine that regulates infiltration of monocytes across the endothelial barrier [55]. CRP also inhibits eNOS activity and bioactivity and NO synthesis and may be involved in further atherosclerosis development [56].

In coronary artery disease (CAD), it is well established that endothelial dysfunction is associated with low-grade inflammation [57]. In PAD, there is also evidence relating endothelial dysfunction to disease severity as well as plasma markers of inflammation. For example, in cultured endothelial cells treated with sera from subjects with PAD, endothelial apoptosis was shown to be 164% greater and endothelial ROS production 62% greater than in cells treated with sera from control subjects without PAD [58]. In addition, the levels of several circulating biomarkers were assessed in the sera of the PAD patients and control subjects, and PAD patients were shown to have lower antioxidant capacity, measured as the Hydroxyl Radical Antioxidant Capacity (HORAC), and higher inflammatory markers of high-sensitivity CRP, interleukin-8 (IL-8), serum amyloid A (SAA), and vascular cell adhesion molecule-1 (VCAM-1) [58]. These data provide yet another link between the prooxidative and proinflammatory status of PAD patients and endothelial dysfunction. 

In two cross-sectional studies, PAD patients with an FMD lower than the 5th percentile of FMD in controls also had significantly increased levels of CRP and fibrinogen, two well-established markers of inflammation [59,60]. Both inflammatory markers were also negatively associated with FMD [59,60]. Likewise, in an analysis of both symptomatic and asymptomatic PAD patients, a positive association between the ABI and FMD and a negative association between the ABI and the expression of the inflammatory markers CRP, ICAM-1, and soluble vascular cell adhesion molecule-1 (VCAM-1) was reported [61]. Furthermore, in patients with concomitant CAD and PAD, the inflammatory markers of neutrophil myeloperoxidase content and plasma levels of interleukin-6 (IL-6) were associated with impaired endothelial function [62]. Thus, the link between endothelial function and inflammatory status and the disease severity in PAD may be a factor explaining the poor prognosis with advanced disease stage. 

Smoking and diabetes mellitus are the strongest risk factors for developing PAD and are both known to promote oxidative stress, which enhances inflammatory pathways [63]. Specifically, oxidative stress can trigger inflammation via activation of the NLRP3 inflammasome and secretion of interleukin-1 (IL-1), activation of nuclear factor kappa-light-chain-enhancer of activated B cells (NF-κB), activation of mitogen-activated protein kinase (MAPK) and secretion of interleukin-8 (IL-8), and by triggering monocyte adhesion [64]. In addition, angiotensin II is thought to induce its actions via generation of ROS and can drive expression of VCAM-1 from endothelial cells [65]. Interestingly, the combined assessment of antioxidant activity, assessed as serum paraoxonase-1 (PON1), an enzyme that protects against lipid peroxidation, and inflammation, measured as monocyte migration by circulating (C-C motif) ligand 2 (CCL2), differentiated PAD patients from controls almost perfectly [66]. 

**Figure 1:**
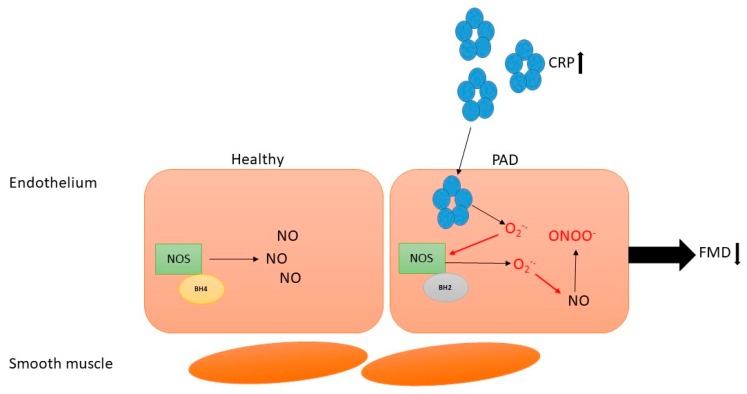
C-reactive protein (CRP) can promote the production of superoxide, which can lead to the destruction of tetrahydrobiopterin (BH4), uncoupling nitric oxide synthase (NOS). This can lead to increased production of superoxide, promoting oxidative stress as well as decreased production and bioavailability of nitric oxide (NO), resulting in endothelial dysfunction, which manifests clinically as reduced flow-mediated dilation (FMD). In PAD patients, CRP and FMD are inversely correlated. Notes. CRP: C-reactive protein, PAD: peripheral artery disease, O_2_^−^•: superoxide, NOS: nitric oxide synthase, BH4: tetrahydrobiopterin, BH2: dihydrobiopterin, NO: nitric oxide, ONOO^-^: peroxynitrite, FMD: flow-mediated dilation.

In PAD patients, CRP and FMD are inversely correlated, and CRP values have also been shown to be higher in patients than in controls and to increase with disease severity [67]. Notably, one factor that may explain the link between endothelial dysfunction and increased oxidative stress and inflammation is NO dysregulation [68]. Specifically, the inducible isoform of NOS (iNOS) is stimulated by cytokines and can produce NO, but it requires the cofactors tetrahydrobiopterin (BH4) and NADPH to work [68]. BH4 can be synthesized from a reduced form of BH2, but oxidative stress impairs this recycling. In fact, when BH4 is depleted, NOS is uncoupled and increases production of superoxide and hydrogen peroxide, reducing NO activity and further increasing oxidative stress [68]. CRP is known to play a role in this process as well, as this acute-phase protein is known to promote the production of superoxide, which leads to the destruction of BH4 and inactivation of NO (Figure 1) [68]. 

Leukocytes are also a major source of ROS, and polymorphonuclear granulocytes (PMN) can produce superoxide by phagocytic NOXs [69]. In fact, PMN are believed to play a role in atherosclerotic development and escalation via increased ROS production and endothelial adhesion [69]. Specifically, PMN interact with platelets by binding with triggering receptor expressed on myeloid cells (TREM)-1, which leads to increased production of ROS by monocytes and PMN [70]. The soluble form of TREM-1 (sTREM-1) is associated with the severity of several inflammatory diseases, and is considered a novel inflammatory marker [71]. PAD patients with critical limb ischemia (CLI) have been shown to have elevated formation of ROS, assessed by chemiluminescence using the luminol analogue L-012, in addition to increased levels of serum sTREM-1 and a higher expression of TREM-1 on PMN [72]. Additionally, sTREM-1 concentrations were correlated with ROS production, and the walking distance of PAD patients was inversely correlated with sTREM-1 [72]. These data support a proposed hypothesis that activated platelets in PAD patients lead to stimulation of PMN and monocytes by the interaction of TREM-ligand with TREM-1, which causes an increase in ROS production and greater platelet activation in a positive feedback loop [9]. 

Different anti-inflammatory agents have been tested in PAD patients due to the increased inflammatory burden associated with the disease. Prostaglandins, a group of active lipid compounds, play an important role in inflammation. Cyclooxygenase (COX) is the enzyme that catalyzes the primary step in the conversion of arachidonic acid into prostaglandins, and it is found in two isoforms, COX-1 and COX-2 [73]. Of the two, COX-2 is present in only atherosclerotic and not healthy arteries and is therefore believed to play a role in plaque formation [74]. One selective COX-2 inhibitor, celecoxib, showed promise as a potential approach to treat early-stage PAD [75]. In patients receiving celecoxib, FMD increased significantly as early as 3 h after the first dose and after 1 week of treatment, with a concomitant decrease in CRP levels [75]. The increase in FMD may be explained by both COX-2 inhibition, since COX-2 is a potent generator of ROS (and ROS can reduce NO bioactivity), as well as the reduction in CRP, which can reduce NO bioavailability [75]. 

## 6. Antioxidants

In theory, attenuation of oxidative stress by the use of antioxidant supplements is promising, although several randomized clinical trials have failed to show any benefit for preventing atherosclerotic progression or preventing the incidence of cardiovascular events [76]. For example, a meta-analysis of 50 trials found no association between supplementation with vitamins and antioxidants and reductions in the risk of major cardiovascular events [77]. Likewise, large-scale trials such as the Heart Outcomes Prevention Evaluation (HOPE) trial showed vitamin E had no effect on cardiovascular outcomes [78]. Additionally, the prevention of progression of arterial disease and diabetes (POPADAD) trial concluded that there was no evidence to support the use of antioxidant therapy in prevention of cardiovascular events and mortality in patients with diabetes and asymptomatic PAD [79]. In contrast, several smaller studies have shown that different antioxidants may be successful treatment strategies in PAD (reviewed in [76]). For example, administration of vitamins C and E was shown to reduce walking-induced oxidative stress in claudicants [80], and treatment with glutathione (GSH) increased pain-free walking distance, macro-circulatory flow after exercise, and post-ischemic hyperemia in Stage 2 PAD patients [81]. Finally, vitamin C was shown to attenuate the augmented exaggerated blood pressure response to exercise of PAD patients by about 50% [82]. 

Several reasons exist as to why ROS-scavenging antioxidants such as vitamins C and E may not be highly beneficial. First, the concentration of antioxidant that would be required to have an effect in the vascular wall may be unachievable [24]. Further, in the context of increased oxidative stress, the vitamins may be rapidly oxidized and depleted to their non-active form, and the reaction of ROS with the antioxidants may also lead to a product that is another type of ROS [24]. Instead, a better strategy may be to prevent formation of the ROS by targeting the enzymes responsible for their formation, such as NOX2. Compounds inhibiting NOX2 have been studied as potential therapeutic targets for cardiovascular disease. For example, gp91dstat, a NOX2-binding peptide acts as a competitive inhibitor of NOX2 assembly by inhibiting p47phox association with gp91phox. Both in vitro and in an in vivo study in mice, gp91dstat was shown to be effective at inhibiting angiotensin II- induced superoxide formation [83]. However, due to the important role NOX2 plays in normal redox signaling, it may be likely that global NOX2 inhibition can lead to deleterious side effects. Furthermore, NOX2 inhibitors may lead to suppressions in normal innate immune responses, as in the case of chronic granulomatous disease [23]. Another molecule that holds more promise as a safe and effective inhibitor of NOX2 is apocynin, which presumably inhibits the binding of p47phox to p22phox [84]. Notably, apocynin is a pro-drug, and its conversion into the active drug form is believed to occur only by interaction with H_2_O_2_ and myeloperoxidase, which is expressed highly only in conditions of enhanced inflammation and oxidative stress, such as vascular disease [23]. However, issues with apocynin include the potential for unspecific effects, and it does not exhibit selectivity for any of the NOX isoforms [84]. The mechanism of action of apocynin is also not yet fully understood, and researchers disagree on whether the molecule’s activity depends on its active metabolite glycoconjugate or diapocynin, which does not seem to be biologically relevant [85]. Further, apocynin shows intrinsic antioxidant activity, and has been suggested to be an ROS scavenger rather than an NOX inhibitor [86]. Interestingly, in humans, apocynin inhalation for 60 and 120 min has been shown to reduce H_2_O_2_ in the breath of healthy subjects [87], asthmatics [88], and COPD patients [89] with no adverse events. Although short-term administration of apocynin is well-tolerated, less is known about whether the pharmacodynamics and pharmacokinetic profiles of the molecule merit long-term administration [23].

In contrast to the global NOX inhibitors, propionyl-L-carnitine (PLC) is a natural short chain l-carnitine ester that has been shown to decrease the activation of NOX, reduce NOX-related superoxide production, and in a rabbit model of hind limb ischemia, PLC accelerated blood flow recovery and increased vascularization [90,91]. PLC has been shown to be efficacious in several settings to counteract NOX-generated oxidative stress-induced inflammation as well [92]. PAD patients in the trial reported by Loffredo et al. received an administration of the antioxidant PLC [21]. Subjects were allocated to receive infusion of either 6 g of PLC or a placebo per day for 7 days in a double-blind cross-over design. Interestingly, FMD and brachial basal diameter significantly increased only in the PLC group and not in the placebo group, accompanied by an increase in NOx and a decrease in 8-OHdG [21]. In another study, Loffredo et al. also demonstrated that serum levels of 8-OHdG were inversely correlated with MWD, and treatment with PLC led to significantly decreased 8-OHdG along with significantly increased MWD and NOx [93]. To identify the mechanism of action of PLC, the translocation of p47phox, which is the phagocyte NADPH oxidase/NOX2 organizer, from the cytosol to the cell membrane of cells was studied [32]. This is a measure of NADPH oxidative activation, since p47phox migrates and binds to the membrane unit NOX2 only after activation [94]. Incubation of agonist-stimulated cells led to an increase of p47phox translocation in the medium as well as increased production of ROS and isoprostanes [32]. However, incubation with PLC led to significant reductions in these changes, suggesting that PLC exerts its antioxidant actions by down-regulation of NOX activation [32].

Polyphenols, naturally occurring compounds that are secondary metabolites of plants, have gained much interest for their importance in human health and disease [95]. Specifically, epicatechin and catechin, natural phenols and antioxidants, seem to play a role in the prevention and treatment of cardiovascular disorders [95]. In the study by Carnevale et al., PAD patients were shown to have enhanced platelet activation, which contributes to alteration of endothelial function, and the supernatant of activated platelets from PAD patients increased the release of soluble cell adhesion molecules (sCAMs) and decreased eNOS activation and NO bioavailability in human umbilical vein endothelial cells (HUVECs) [96]. However, pretreatment with epicatechin and catechin reduced this endothelial activation by decreasing sCAMs and increasing eNOS activity and NO bioavailability [96]. Likewise, 40 g of dark chocolate, which is known to be a NOX2-specific antioxidant, has been shown to acutely increase MWD, maximal walking time (MWT), and serum NOx in PAD patients, with a concomitant decrease in NOX2 activity and serum isoprostanes [97]. 

Ischemia by acute exercise has been suggested to reduce FMD in PAD patients [17]. However, Silvestro et al. separated patients into maximal and submaximal exercise groups, and only maximal exercise was shown to reduce FMD in PAD patients, with no changes in FMD observed when patients were asked to walk until the onset of claudication pain [98]. Furthermore, the decreased FMD that accompanied maximal exercise was accompanied by an increase in plasma levels of thiobarbituric acid-reactive substances (TBARS), a measure of oxidative stress by lipid peroxidation, as well as sCAM-1 [98]. However, when patients received an infusion of 50 mg/min of vitamin C for 20 min prior to exercise, there were no decreases in FMD or elevations in TBARS and sCAM-1, even following maximal exercise [98]. Together, these data suggest potential important functional and clinical implications of antioxidants, emphasize the role of endothelium-derived vasodilation on walking performance, and reinforce the proposed mechanism of oxidative-stress mediated arterial dysfunction in PAD patients.

## 7. Discussion

PAD is associated with cardiovascular events as well as severe functional impairment and decline. Individuals with PAD have poorer walking endurance than individuals without the disease, and this walking endurance also decreases with increasing disease severity [99]. In fact, even asymptomatic PAD patients exhibit functional impairment and functional decline [99]. Impairments in functional performance are associated with cardiovascular events as well as poor quality of life [8]. Measures of endothelial function, such as FMD, and measures of arterial stiffness, including PWV and AIX, are likely players that affect functional performance in PAD patients [8]. Importantly, these measures are also associated with mortality and cardiovascular events in these patients [8]. Further, assessment of endothelial function and arterial stiffness may become valuable tools in determining the severity and extent of atherosclerosis in PAD as well as in identifying high-risk patients [50]. 

Interestingly, there is a growing amount of evidence that suggests that NO is involved in endothelial dysfunction, arterial stiffness, and inflammation, as well as the oxidative stress that leads to atherosclerotic development in PAD [67]. Vascular homeostasis is maintained under normal physiological conditions, but oxidative stress can lead to activation of endothelial cells that causes atherosclerotic lesion progression [50]. The endothelium maintains its protective function against vascular disease mainly by NO production, NO-mediated relaxation of vascular smooth muscle cells, and NO-mediated inhibition of smooth muscle cell hypertrophy and activation of platelets [23]. Oxidative stress, which is a hallmark of PAD, is known to reduce vascular NO bioavailability. Specifically, the NOX enzymes are implicated as the main source of endothelial and vascular ROS production [23]. In normal physiology, these ROS are important for redox signaling, but in diseased states, NOX activity in the vascular wall is upregulated, which can contribute to enhanced oxidative stress [23]. Out of the four endothelial NOX enzymes, NOX2 is believed to hold the greatest implication for vascular disease by NOX-2 derived ROS inactivation of NO [23]. PAD may be a diseased state in which endothelial NOX2 expression is upregulated, as PAD patients have been shown to have enhanced levels of markers of NOX2 activation compared to controls [32]. This can be explained by the fact that NOX2 expression and activity seem to be upregulated following ischemia, and NOX2 actually plays a significant role in ischemia reperfusion injury, which occurs when blood supply returns to ischemic tissue [100].

It is important to note that a majority of NO is generated in the cytosol, so for NOX2-derived ROS to interact with NO, it must first enter the cytosol or extracellular compartments [23]. However, the primary sites of NOX2 activity are the endoplasmic reticulum and nuclear membranes; therefore, most of the NOX2-derived ROS, which cannot freely cross membranes, will theoretically remain within those subcellular compartments [23]. In addition, the vascular wall is known to express high levels of the antioxidant enzyme superoxide dismutase (SOD), which can convert superoxide into less damaging species [23]. However, evidence suggests that antioxidant defenses, including SOD, are compromised in PAD [101]. It has also been shown that NOX2-derived ROS production in the extracellular space is in fact increased in vascular disease [23]. Atherogenic molecules such as tumor necrosis factor α (TNF-α), cholesterol, and homocysteine, which have all been shown to be significantly elevated in PAD [102,103], increase endothelial NOX2 expression [23]. Furthermore, these molecules lead to clustering of lipid raft domains, which causes NOX2 aggregates to form in the plasma membrane [23]. This in turn leads to high levels of ROS being produced in the extracellular space that can interact with NO [23]. 

NOX2 inhibitors have gained interest as novel therapeutics for vascular disease, but issues related to innate immune suppression and other potential deleterious side effects have limited in vivo assessments. Specifically, there is a lack of data supporting the suitability of long-term NOX2 inhibition [23]. However, PLC has been widely studied in PAD, and has been reported to improve the muscle carnitine deficiency characteristic of PAD, improve walking capacity, and enhance most quality of life measures, leading the Trans-Atlantic Inter-Society Consensus II to recommend its use to improve symptoms associated with PAD [104]. A meta-analysis by Brass et al. of PLC effects on performance in PAD patients concluded that PLC is associated with a statistically significant increase in walking distance that may also be clinically relevant [105]. The studies included in this review suggest that reduced NO synthesis and activity in PAD may be the result of enhanced oxidative stress, and this in turn may lead to endothelial dysfunction, arterial stiffness, and inflammation. Administration of PLC is associated with enhanced endothelial function, improved NO synthesis, a reduction in oxidative stress, and an improvement in walking distance, which seems to be mediated by NOX2 inhibition [21,32,93]. Likewise, dark chocolate was shown to improve walking autonomy in PAD patients, presumably by down-regulation of NOX2-mediated oxidative stress [68]. 

## 8. Conclusions

Evidence suggests that endothelial dysfunction and arterial stiffness may be implicated in PAD. Specifically, several studies have shown that endothelial dysfunction and arterial stiffness are related to cardiovascular risk in patients with PAD. Notably, both of these processes are linked to oxidative stress and inflammation. Data from several research groups have shown that oxidative stress markers are elevated in PAD patients, in correlation with measures of endothelial dysfunction and arterial stiffness. One mechanism that may explain this link is based on decreased production and bioavailability of NO in response to elevated levels of ROS. Furthermore, it is likely that up-regulation or over-activation of NOX2 is a major source of these ROS in PAD patients. Therefore, targeting NOX2 by specific antioxidants or inhibitors may play a role in improving arterial function and functional status in PAD patients. Future studies should assess the long-term effects of PLC and dark chocolate administration to determine if they may be novel approaches for PAD treatment.
